# On the Internal Use of Veratrum Viride

**Published:** 1852-03

**Authors:** 


*On the internal use of Veratrum Viride.*—In our last issue we copied an article by Dr. Norwood relative to the medicinal properties of veratrum viride. The article bore internal evidence of great extravagance in the ideas of the writer respecting the powers of his favorite remedy; but thinking that there might be some foundation for his enthusiasm, we were desirous that the subject should be brought to the notice of our readers, and we shall be glad to hear from any who may have facts pertaining thereto.

The following is a portion of an article in the Charleston Med. and Surg. Journal, containing an account of the doses, and mode of administration together with the results of a limited trial of the remedy by some of the physicians of Charleston.—EDITOR BUFFALO MED. JOUR.

The preparation used by Dr. Norwood, is the saturated tincture of the root.

The doses, as recommended by Dr. Norwood are as follows:

| | | | | | | | |
|---|---|---|---|---|---|---|---|
| Children | from | 2 | to | 3 | years, | 2 | drops. |
| " | " | 4 | " | 6 | " | 3 | " |
| " | " | 7 | " | 10 | " | 4 | " |
| " | " | 11 | " | 15 | " | 5 | " |
| Ladies and Lads | of | 18 | | | " | 6 or 7 | " |
| Adult males | | | | | | 8 | " |

Repeat the dose every two or three hours, in all ages and cases.

When the pulse is sufficiently reduced or nausea or vomiting occurs, reduce the quantity one-half in all cases, keeping up the period of administration. If there is more nausea and vomiting than desirable, a little brandy and laudanum, or tincture of ginger and syrup of morphine. If the first portion of the brandy, laudanum or morphine, should fail to relieve, repeat it. Intense paleness and coolness or coldness, also nausea and vomiting, are some of its leading effects. The great and important effect is in the reduction of the frequency of the pulse.

Such are the pretensions with which this remedy has been heralded forth to the medical public. It remains to be seen whether its reputation will be sustained by the general voice of the profession, or whether the encomiums lavished upon it are extravagant and unmerited.

In this city, (Charleston) the trials made with it have been too few in number to warrant us in drawing any conclusion in reference to its remedial powers. It has been exhibited by one of the editors of this journal, and by one or two other physicians in typhoid fever, pneumonic congestion, &c.; and although it promptly reduced the force and frequency of the pulse, it had no perceptible effect upon the progress of the disease.

